# The association of parental temperament and character on their children’s behavior problems

**DOI:** 10.7717/peerj.1464

**Published:** 2015-12-01

**Authors:** Soo Jin Lee, C. Robert Cloninger, Soo Hyun Park, Han Chae

**Affiliations:** 1 Department of Psychotherapy, School of Nursing and Public Health, Kyungil University, Daegu, Korea; 2 Department of Psychiatry, School of Medicine, Washington University in St. Louis, St Louis, MO, USA; 3 Department of Psychology, College of Liberal Arts, Yonsei University, Seoul, Korea; 4 School of Korean Medicine, Pusan National University, Busan, Korea

**Keywords:** Temperament and character, Children’s problem behavior, Personality development, Parent–child relations

## Abstract

**Purpose.** Parents have important roles in child rearing, but the influence of their personality on rearing practices and their impact on the behavior of children has received surprisingly little attention. The aim of the current study was to investigate the relationship between parents’ personality and children’s problem behaviors.

**Materials and Methods.** Participants consisted of 190 preschool outpatients (104 boys, 86 girls) and their parents who visited traditional Korean pediatric clinics with minor physical symptoms as chief complaints. The personality profiles of the both parents were measured by the Temperament and Character Inventory and children’s behavior problems by the Child Behavior Checklist 1.5–5. Correlation and stepwise regression analysis were employed for the statistical analyses.

**Results.** The temperament trait of Harm Avoidance and the character traits of Self-Directedness and Self-Transcendence of the parents were significantly correlated with children’s problem behaviors. Character as well as temperament, played an important role in explaining children’s problem behaviors after age and gender of children were taken into account.

**Conclusion.** The maturity of parents’ character appears to have a key role in reducing the risk of behavior problems in their children. Suggestions are made for parental education and future research.

## Introduction

There has been extensive research examining the relationship of family environment and parenting on the adjustment of children (Baumrind, 1967; Bowlby, 1969). In particular, it has been well documented that parental abuse and neglect have a negative impact on the adaptation of children (Patterson, DeBaryshe & Ramsey, 1989). In addition, psychopathology in parents, such as depression in mothers (Downey & Coyne, 1990; Goodman & Gotlib, 1999) or emotional distress of parents (Anthony et al., 2005; Õstberg, 1998) are predictive of their children’s problem behaviors. The relations between the temperaments of parents and their children have also been shown to influence the risk of children’s problem behaviors (Lee, 2012; Rettew et al., 2006). That is, they investigated the interaction of temperament between children and parents in order to explain the children’s problem behaviors such as the association between mother’s HA and children’s externalizing problem behaviors or father’s HA and children’s internalizing problem behaviors.

However, few studies have directly studied the role of the maturity and integration of parents’ personality on the risk of their children’s behavior problems: the studies provided above have not included the parent’s character dimensions with temperament dimensions (Josefsson et al., 2013a). An individual’s personality involves more than their temperament; it includes the way a person regulates his or her goals and values to achieve a long-term purpose, such as rearing healthy children. Some studies have described the relationship between parents’ personality and specific child problem behaviors, such as antisocial behaviors and depression (Bates et al., 1991; Brenning et al., 2011; Davies et al., 2012; Nigg & Hinshaw, 1998). That is, most of the past studies have measured abnormal traits in parents, and have not distinguished temperament and character of parents. Therefore, more research is needed about the role of healthy character traits in parents and its impact on children’s behavior problems.

Cloninger’s psychobiological theory of personality (Cloninger, 2008) postulates that personality is composed of temperament and character, two-interrelated domains which are hypothesized to interact as a non-linear dynamic system in regulating the development of human psychological functions. Temperament and character are considered to interact dynamically in the development of personality across the lifespan (Cloninger, Svrakic & Svrakic, 1997). Temperament traits reflect biases in automatic responses to emotional stimuli involving involuntary rational processes, whereas character traits depict differences in higher cognitive functions underlying a person’s goals, values, and relationships (Cloninger, 2008; Cloninger, Svrakic & Przybeck, 1993).

Character influences the meaning that is given to events as a result of one’s goals and values. As a result, differences in the maturity and integration of a person’s character can modify a person’s emotional reactions and allow self-regulation of emotions and behavior (Cloninger et al., 1994a). Consequently, character traits are more predictive than temperament traits of a person’s level of well-being or their level of psychological disorder (Cloninger & Zohar, 2011). Even when people have similar temperament profiles, it is likely that differences in the maturity and integration of their character will influence the quality of the rearing environment they provide for their children.

The aim of the current study is to investigate the relationship between the personality characteristics of parents and their children’s problem behaviors. Furthermore, we sought to distinguish the roles of temperament and character in parents on the risk of behavior problems in their children in order to identify those aspects of personality that have the greatest impact on effective child rearing.

## Materials and Methods

### Participants

Participants were recruited from outpatients who visited specialized traditional Korean pediatric clinics in the suburban Seoul metropolitan area with minor physical symptoms (i.e., common cold, and rhinitis/sinus infection etc.) as chief complaints. The primary care doctors suggested participation in the research with detailed explanation to the parents of preschoolers aged between 3 and 6 years. That is, the participation of the parents was purely of their own volition and there would be no consequence to their current or future treatment at the clinics. After the participants had completed a written consent form, the mothers completed both the Child Behavior Checklist (CBCL) and Temperament and Character Inventory (TCI) and the fathers, who often did not accompany with children, completed the TCI only. They were also asked to rate the Socio Economic Status (SES) and education in the extra items of questionnaire. This study was approved by the Department Research Review Committee of the Department of Psychology, Yonsei University (DRC-2009011024).

The demographic characteristics of child patients and their parents are shown in Table 1. The age and gender distribution of the 190 children were not significantly different (${\chi }_{(4,N=190)}^{2}=3.046$, *p* = .550) and no significant differences in distribution were observed between Socio Economic Status (${\chi }_{(4,N=190)}^{2}=3.327$, *p* = .505) and parent education (${\chi }_{(4,N=186)}^{2}=.937$, *p* = .626 for father; ${\chi }_{(4,N=186)}^{2}=.860$, *p* = .651 for mother) with gender of children.

**Table 1:** Demographic characteristics of child outpatients and their parents.

Demographic variables	Boys (*n* = 104)	Girls (*n* = 86)	Totals (*n* = 190)
Children			
Age (months)	56.95 ± 13.07	56.09 ± 12.85	56.56 ± 12.94
Mothers (*n* = 186)			
Age (years)	34.37 ± 2.70	35.56 ± 4.26	34.91 ± 3.54
Education			
High school (%)	6 (10.9)	8 (9.4)	14 (7.5)
College (%)	84 (83.2)	69 (81.2)	153 (82.3)
Master degree (%)	11 (5.9)	8 (9.4)	19 (10.2)
Fathers (*n* = 186)			
Age (years)	36.52 ± 2.85	37.60 ± 3.06	37.01 ± 2.99
Education			
High school (%)	7 (6.9)	8 (9.4)	15 (8.1)
College (%)	66 (65.3)	58 (68.2)	124 (66.7)
Master degree (%)	28 (27.7)	19 (22.4)	47 (25.3)
Socio economical status (*n* = 170)			
Upper class	1 (1.1)	0 (0)	1 (0.6)
Upper middle class	15 (16.3)	12 (15.4)	27 (15.9)
Middle class	50 (54.3)	50 (64.1)	100 (58.8)
Low middle class	22 (23.9)	15 (19.2)	37 (21.8)
Low class	4 (4.3)	1 (1.3)	5 (2.9)

**Notes.**

Results are reported as means ± standard deviation or as frequency (%).

### Assessments

The Korean version of the Temperament and Character Inventory-Revised-Short (TCI-RS) (Min, Oh & Lee, 2007) is a 140-item self-report questionnaire, which asks individuals to rate each item on a 5-point scale (0 = not at all to 4 = very true). The Korean version of the questionnaire was standardized and validated in 2007 and demonstrated good validity and reliability (Min, Oh & Lee, 2007).

The temperament dimensions consist of Novelty Seeking (NS, characterized by exploratory excitability, impulsiveness, extravagance, and disorderliness), Harm Avoidance (HA, anticipatory worry, fear of uncertainty, shyness with strangers, and fatigability), Reward Dependence (RD, sentimentality, openness, attachment, and dependence), and Persistence (PS, eagerness, work-hardened determination, ambition, and perfectionism). The three dimensions of character consist of Self-Directedness (SD, responsibility, purposefulness, resourcefulness, self-acceptance, and congruent second nature), Cooperativeness (CO, social acceptance, empathy, helpfulness, compassion, and principled), and Self-Transcendence (ST, self-forgetfulness, transpersonal identification, spiritual acceptance) (Cloninger et al., 1994b). Cronbach’s alphas for the NS, HA, RD, PS, SD, CO, and ST scales were 0.83, 0.86, 0.81, 0.82, 0.87, 0.76, and 0.90, respectively (Min, Oh & Lee, 2007).

The children’s problem behavior was measured using the Korean version of the CBCL for ages 1.5–5 (Oh & Kim, 2008), a questionnaire consisting of 100 items developed for measurement of problem behavior in the children. Parents were asked to rate the behavior of the child for the preceding six months on a three-point Likert scale (0 = not true at all, 1 = somewhat or sometimes true, and 2 = very true or often true). The Korean version of CBCL was standardized and validated in 2008 and demonstrated good validity and reliability (Oh & Kim, 2008). During the process of standardization in Korea, children of age 6 who were enrolling in kindergarten were included, which enabled use of the CBCL in six-year-old children.

The CBCL yields three subscales of Total score, Internalizing Problems and Externalizing Problems, and eight empirically-validated symptom clusters including Emotionally Reactive, Anxious/Depressed, Somatic Complaints, Withdrawn, Sleep Problems, Attention Problems, Aggressive Behavior, and Other Problems. Internalizing Problems includes Emotionally Reactive, Anxious/Depressed, Somatic Complaints, and Withdrawn, Externalizing Problems includes Attention Problems and Aggressive Behavior, and Total Problems includes Internalizing Problems, Externalizing Problems, Sleep Problems, and Other Problems. Cronbach’s alphas for the internalizing, externalizing, and total problem behaviors of the present study were .88, .90, and .86, respectively.

### Statistical analysis

We examined the age and gender distribution using chi-square analysis and explored the relationship between subscales of parental TCI and CBCL with correlation analysis. Two series of multiple stepwise regression analyses were then performed in order to determine whether the parental TCI traits could explain the problem behaviors of children. First, all personality variables were introduced and their significance in explaining the contribution of each problem behavior was assessed (model 1). Second, age and gender measures were added (model 2). Results are shown as means ± standard deviations or as frequency (%). All analyses were performed using IBM SPSS Statistics 20.0 for Windows (IBM, Armonk, NY, USA) and *p* values of 0.05, 0.01, and 0.001 were used for significance.

## Results

The internalizing and externalizing subscales of the CBCL were moderately correlated with one another (*r* = .66) and strongly correlated with total problems: the correlations with total problems were 0.91 for internalizing problems and 0.88 for externalizing problems.

The correlations between temperaments of mother and father and children’s problem behaviors using TCI and CBCL Internalizing, Externalizing, and Total Problem behaviors are shown in Table 2. All three internalizing, externalizing, and total problems showed positive correlations with the scores for HA (Harm Avoidance) of both parents. All three problem scores were also correlated positively with the mother’s scores for ST (Self-Transcendence) and negatively with the mother’s score for SD (Self-Directedness). Internalizing, Externalizing, and Total Problem behaviors in children were not correlated with the temperaments except for HA. Likewise, none of the problem behaviors were correlated with the CO (Cooperativeness) of the mother.

**Table 2:** Correlation coefficients among children’s problem behaviors and TCI scales of parents.

	Mother							Father						
	NS	HA	RD	PS	SD	CO	ST	NS	HA	RD	PS	SD	CO	ST
Children’s behavior problems														
Total	.11	**.21** ^**^	.09	−.07	**−.38** ^**^	−.04	**.19** ^**^	−.04	**.21** ^**^	−.04	−.05	−.07	−.07	−.09
Internalizing	.08	**.23** ^**^	.08	−.05	**−.33** ^**^	−.05	**.18** ^**^	−.06	**.18** ^*^	−.04	−.04	−.03	−.08	−.13
Externalizing	.11	**.16** ^*^	.06	−.09	**−.36** ^**^	−.01	**.16** ^*^	.01	**.16** ^*^	−.02	−.01	−.04	−.03	−.07

**Notes.**

* *p* < 0.05.

** *p* < 0.01.

Bold represents significance.

Total total problems of CBCL Internalizing internalizing problems of CBCL Externalizing Externalizing problems of CBCL NS Novelty Seeking HA Harm Avoidance RD Reward Dependence PS Persistence SD Self-Directedness CO Cooperativeness ST Self-Transcendence

To further evaluate the associations shown in Table 2, stepwise regression analyses were performed on each of the problem behaviors. First, all seven personality variables were introduced in the model (model 1). Second, demographic variables including age and gender were added to this model 1 (model 2). Table 3 reported here was the model 2 after all seven personality traits, age and gender were all included. We found that Total Problem behavior was associated with greater HA and ST and lower SD of mother; that is mothers who reported more problems in their children were anxious and tended toward unrealistic high expectations of their children (i.e., low SD and high ST). In contrast, the fathers of children with problem behaviors were high only in HA (i.e., they were anxious but near average in character). The addition of the covariates for sex and age did not modify the results for Total Problems. The result for Internalizing Problem behavior was similar to that for Total Problems, as expected given their strong correlation. However, for Externalizing Problem behavior, ST was not a significant predictor in the multiple regression equation (see Table 3).

**Table 3:** Stepwise regression analysis between children’s problem behavior and TCI of parents.

	95% Confidence interval for unstandardized coefficient (*B*)		Standardized coefficient	*t*
	Lower bound	Upper bound	*β*	
Total problem				
SD (mother)	−1.018	−.732	−.394	−6.11
HA (father)	.551	.919	.375	3.988
SD (father)	.315	.739	.236	2.483
ST (mother)	.252	.506	.189	2.828
ST (father)	−.507	−.237	−.184	−2.766
	*F*(5, 181) = 12.820, *p* < .001, adj. *R*^2^ = .241			
Internalizing problem				
SD (mother)	−.354	−.240	−.348	−5.26
HA (father)	.197	.343	.358	3.712
SD (father)	.134	.302	.254	2.602
ST (father)	−.219	−.113	−.213	−3.136
ST (mother)	.085	.189	.180	2.632
	*F*(5, 181) = 10.466, *p* < .001, adj. *R*^2^ = .203			
Externalizing problem				
SD (mother)	−.345	−.241	−.377	−5.589
HA (father)	.152	.286	.319	3.262
SD (father)	.104	.258	.231	2.358
	*F*(3, 183) = 13.313, *p* < .001, adj. *R*^2^ = .166			

**Notes.**

HA Harm Avoidance SD Self-Directedness ST Self-Transcendence

## Discussion

The current study investigated the relationship between parental personality and children’s problem behaviors. For this purpose, we used the TCI to assess the temperament and character aspects of the parents’ personality. We found that lower scores on Self-Directedness and higher scores on Harm Avoidance and Self-Transcendence were associated with greater maternal reports of their children’s problem behaviors. The parental profiles associated with greater reports of children’s problem behaviors were characteristic of parents who have high expectations of themselves and of those who they perceive to be extensions of themselves. To understand the significance of the observed parent–child relationships, it is important to understand the nature of the personality measures and how they influence behavior.

The mothers who reported more behavior problems in their children were anxious (i.e., high in Harm Avoidance) and they had a tendency toward unrealistically high expectations of their children (i.e., they were low in Self-Directedness and high in Self-Transcendence). The fathers of children with problematic behaviors were also anxious (i.e., high in Harm Avoidance) but they were not low in Self-Directedness. The zero-order correlation of the father’s Self-Directedness was negligible, but in the multiple regression analysis, higher Self-Directedness of the father contributed to the risk of the mother reporting more problems in the children. This suggests that possible interpretation that the discrepancy between the Self-Directedness of the two parents increased the mother’s expectations and demands on the children. When a father is a hard-working wage-earner who has little time to help the mother or the children, as is common in Korea, the greater Self-Directedness of the father may contribute to family conflict and children’s problem behaviors. In other words, the higher Self-Directedness of the father may be an indicator of his absence from the home because of his over-commitment to work; as a result, he is unlikely to be helpful in child-rearing, which leads to problems when the mother is not a calm, resourceful caretaker herself.

Mothers who are low in Self-Directedness and high in Self-Transcendence can be characterized as a having magical and unrealistic thinking; in other words, they become preoccupied by their fantasies and wishful expectations rather than accepting realistic facts and being practical (Cloninger et al., 1999). Such a profile results in ignoring and underestimating signs of children’s physical and psychological symptoms as a result of magical thinking or maintaining unrealistic goals and impractical expectations for a child. Such unrealistic mothers are likely to report many problems in their child because they have unrealistic expectations of the child. It is also noteworthy and interesting that externalizing problems were associated with higher Harm Avoidance and lower Self-Directedness in both parents, but were not associated with Self-Transcendence in either parent. This difference suggests the possibility that an absent worker-father in Korean families may really increase the risk of their children’s delinquency, especially when the mother is not self-directed.

The novel finding of the current study is the demonstration that the character of parents can play an important role in predicting problem behaviors of children. We found that parental temperament, particularly anxiety-proneness (Harm Avoidance) does have a role, but the character profiles of the parents has a substantial impact on the risk of problem behaviors regardless of the effect of temperament. This finding supports the traditional concept that calm and mature parents who are engaged in their children’s lives can help children to be well-adjusted. Such mature parenting style considered as self-disciplined but sensitive and responsive would be indicated by TCI profiles of being highly developed in all the character scales or at least in Self-Directedness and Cooperativeness (Josefsson et al., 2013a). However, weak development of Self-Directedness and Cooperativeness or excessive development of Self-Directedness without Cooperativeness to facilitate engagement with the family may interfere with parents being effectively engaged as role models for their children. Mature and effective parenting may also be measured by parent’s being low in neuroticism, high in extraversion, agreeableness, openness to experience and conscientiousness, as well as high in self-esteem and internal locus of control (Belsky & Barends, 2002). The findings of Josefsson and his colleagues (2013b) that parental care-giving and home-environment predicted the character traits and not the temperament traits of their children also support the current results. Together these findings suggest that maturity of parents’ character has a stronger impact on child development than does the emotional style of the parents.

Our findings are comparable to the authoritative parenting described by Baumrind (1966). According to her, “the authoritative parent shares the reasoning with child behind her policy …autonomous self-will and disciplines conformity is valued by the authoritative parents …(she) exerts firm control at points of parent–child divergence but does not hem the child in with restrictions. She uses reason, power, and shaping by regime and reinforcement to achieve her objectives.” This definition emphasizes the importance of more sensitive, responsive, supportive and intellectually stimulating parenting or mature parenting focusing on cognitive, conscious, voluntary control rather than emotional, prompt, involuntary emotional reactivity to environmental stimuli in caregivers. Such authoritative parenting is also comparable to mindfulness training, which has been shown to promote character development as measured by the TCI when practiced regularly (Campanella et al., 2014). The effects of long term social relationships on the maturation of character is also shown by gradual convergence in the character traits of spouses in stable long-term partnerships in Korea (Yang et al., 2015). In order to generalize these results, future study should be conducted in the general population of children and other different age groups considering the fact that the current data comes from an outpatient clinic with a higher level of education and SES. Second, the lack of independent measure of child behavior problems can affect the conclusion resulting from the common method variance (Podsakoff et al., 2003). Therefore, independent measure of child behavior problems should be added in the future study. Third, the parental practices should be included as potential explanatory variables in order to determine if temperament and character of parents are more important variable than others. Fourth, there are cultural and social differences between American and Korean children in the role of attachment on children’s problem behaviors. In American but not Korean children, more frequent problem behaviors were observed in children of mother with unstable attachment style than in children of mothers with a stable attachment style (Han, 2002). Therefore, future study using subjects with different cultural backgrounds should be conducted in order to better understand the role of parental personality in the complex processes that influence child development.

## Supplemental Information

10.7717/peerj.1464/supp-1 Supplemental Information 1 Raw data for the manuscript

## Additional Information and Declarations

### Data Availability

The following information was supplied regarding data availability: Raw data can be found in the Supplemental Information.
